# Multiple facial candidal abscesses after self-administered acupuncture in a patient with undiagnosed diabetes mellitus: a case report

**DOI:** 10.1186/s12906-021-03343-w

**Published:** 2021-06-10

**Authors:** Jae Yun Sung, Ju Mi Kim, Jong Uk Lee, Yeon Hee Lee, Sung Bok Lee

**Affiliations:** 1 Department of Ophthalmology, Chungnam National University Sejong Hospital, Sejong, Republic of Korea; 2 Department of Ophthalmology, Chungnam National University College of Medicine, Chungnam National University Hospital, #282 Munhwa-ro, Jung-gu, Daejeon, 35015 Republic of Korea

**Keywords:** Case report, *Candida albicans*, Facial abscess, Fungal abscess

## Abstract

**Background:**

Facial abscess caused by *Candida albicans* infection is a rare condition even in immunocompromised patients, and only a few cases have been reported. To our knowledge, this is the first case of multiple facial candidal abscesses caused by self-administered acupuncture in an undiagnosed diabetes mellitus patient.

**Case presentation:**

A 57-year-old woman who had self-acupuncture treatment 2 weeks previously, presented with a 1-week history of progressive left eyelid swelling, erythema, and pain. Despite the antibiotic treatment, the lesion progressed. Surgical incision and drainage was performed and *Candida albicans* was isolated from the obtained pus culture. The patient was diagnosed with type 2 diabetes mellitus based on a random serum glucose level of 350 mg/dl and 9.2% HbA1c. The abscess resolved after seven incision and drainage cycles and 4 weeks of intravenous fluconazole treatment with an appropriate control of diabetes mellitus.

**Conclusion:**

Unusual organisms and underlying immunocompromised condition should be suspected in cases of recurrent abscess showing an inadequate response to antibiotic treatment.

## Background

*Candida albicans* (*C. albicans*) is an opportunistic fungal pathogen found in normal microflora. As a commensal, *C. albicans* asymptomatically colonizes skin and mucosal surfaces, however, under certain circumstances, *C. albicans* can proliferate and invade causing infections ranging from mucocutaneous infection such as oral or vaginal candidiasis to systemic infections such as candidemia or disseminated candidiasis [1]. Mucocutaneous candidiasis is usually self-limiting in immunocompetent hosts and can be easily treat with local treatment, however, in immunocompromised patients, it can be a gateway to systemic spread. Disseminated candidiasis is a devastating disease associated with high morbidity and mortality rates. Population-based surveillance studies report the yearly incidence of *Candida* infections as 8 per 100,000 populations [2]. Recently, new antifungal agents and new therapy strategies resulted in the alteration of *Candida* species causing invasive infections [2, 3].

Facial abscess caused by *C. albicans* infection is a rare condition even in immunocompromised patients and only a few cases have been reported [4–7]. Candidal infection of subcutaneous tissue may result from direct contact, inoculation injury or hematogenous spread [8].The authors report a case of multiple facial candidal abscesses caused by self-administered acupuncture in an undiagnosed diabetes mellitus patient.

## Case presentation

**Fig. 1:**
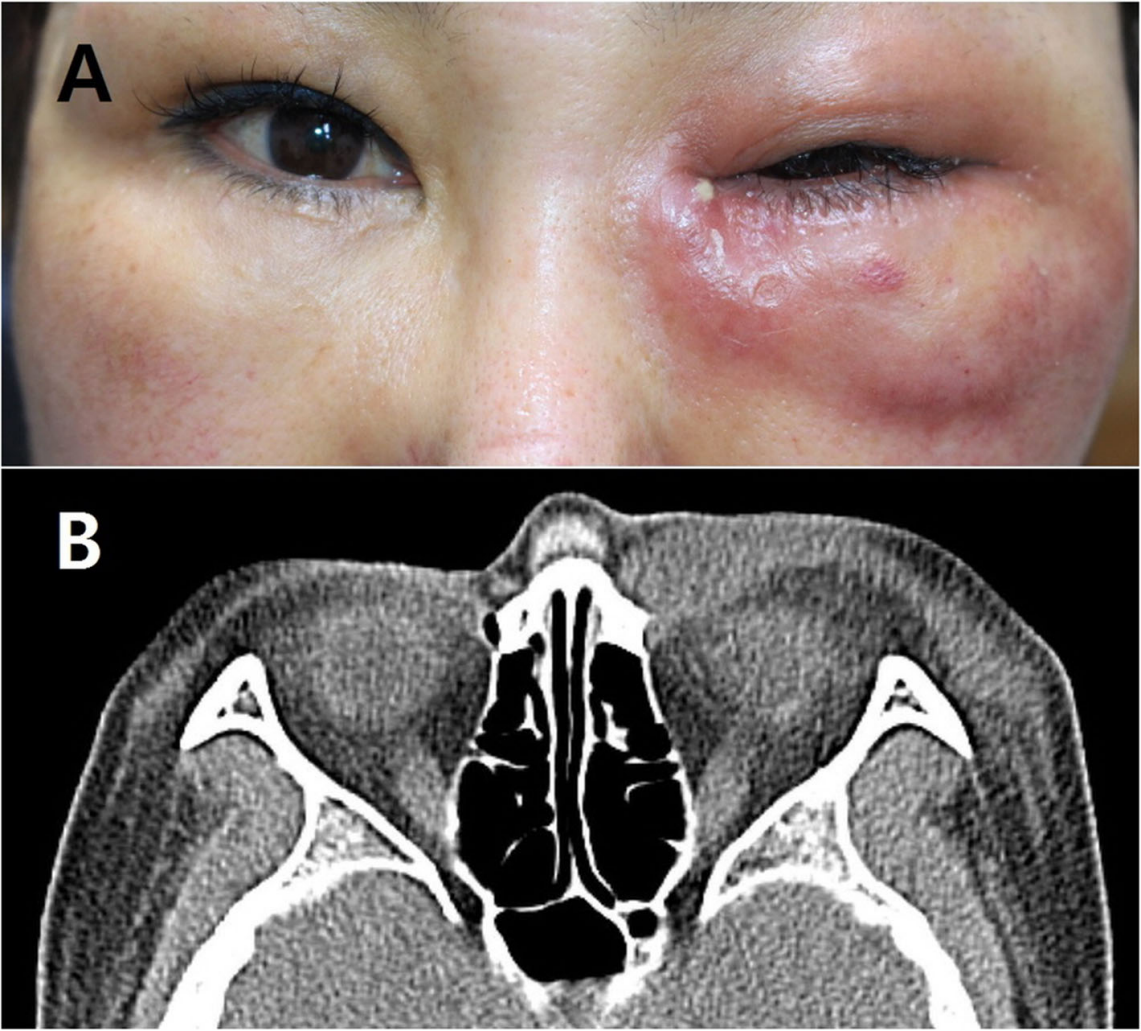
**A** External photograph showing swelling and erythema of the left eyelid, **B** facial computed tomography image showing soft tissue swelling in the left periorbital area

A 57-year-old woman presented with a 1-week history of progressive left eyelid swelling, erythema, and pain. The patient reported a history of blunt eyelid trauma without skin damage 2 weeks prior. She had no significant medical history and was not on any routine medications. On ophthalmic examination, her best-corrected visual acuity was 20/20, in both eyes. Her intraocular pressure, extraocular movements, and pupillary reaction were within normal limits, in both eyes. External examination revealed swelling of the left upper and lower eyelids with erythema and warmness (Fig. 1A). In addition, fluctuation and tenderness were present on palpation. Because small skin wounds were observed in the center of the swollen lower eyelid, the patient’s history was reviewed again. She mentioned that she made punctures at specific points on the lower eyelid, known as acupuncture points to try to treat the eyelid swelling and bruising that had occurred after trauma. She self-administered acupuncture at home using a non-disposable needle without an adequate skin disinfection.

**Fig. 2:**
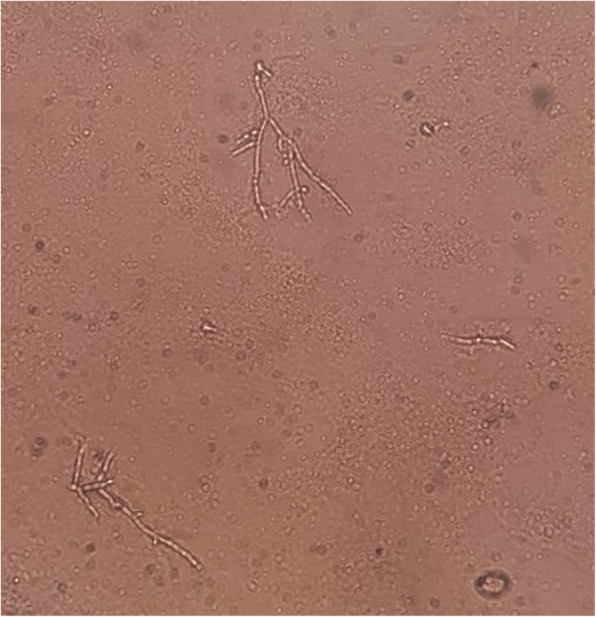
KOH preparation of the pus, showing branched hyphae and budding yeast

Computed tomography images showed soft tissue swelling in the left periorbital area (Fig. 1B). No orbital fracture was identified. The clinical manifestations and radiologic findings were consistent with abscess. The patient was admitted and intravenous antibiotics (metronidazole, ceftriaxone, and amoxicillin) were started. Despite the treatment, the lesion progressed. On hospital day 2, incision and drainage was performed and a KOH smear with pus culture was conducted*. C. albicans* was isolated and a final diagnosis of facial candidal abscess was made (Fig. 2). The laboratory test revealed 350 mg/dl random serum glucose level and 9.2% of hemoglobin A1c. An additional workup of complement and immunoglobulins was normal. A consultation was scheduled at the Department of Endocrinology and she was diagnosed with type 2 diabetes mellitus.

For the treatment of facial candidal abscess, 400 mg intravenous fluconazole was prescribed once a day. During the admission period, the patient underwent five more incision and drainage cycles due to recurrence and the development of new lesions. While maintaining the antifungal treatment and diabetes mellitus management, the lesion gradually improved. Her serum glucose level was well controlled using insulin. After 3 weeks of treatment, the patient’s symptoms and signs resolved, thus, the intravenous fluconazole was changed to oral fluconazole. However, on the third day, the lesion recurred. She underwent incision and drainage followed by 1 additional week of intravenous fluconazole treatment, she was then discharged. Oral fluconazole was given as a maintenance medication for 4 weeks. There was no recurrence at the 6-month follow-up.

## Discussion and conclusions

*C. albicans* is an opportunistic fungal pathogen found as part of the normal microflora in human skin, and in intestinal and genital mucosa. However, under certain circumstances, *C. albicans* can cause infections ranging from non-life-threatening mucocutaneous infections to life-threatening systemic infections. Risk factors promoting infection include immunodeficiency, malignancy, diabetes mellitus, prolonged antibiotic therapy and corticosteroid use [1, 9, 10]. Facial abscess caused by *C. albicans* infection is rare even in immunocompromised patients [8]. In the literature review, only 4 cases have been reported previously.

Costin et al. [4] reported recurrent *C. albicans* eyelid abscess in a 1-year-old child with severe neutropenia. Chen et al. [5] described facial *C. albicans* cellulitis that occurred 2 months after steroid injection treatment in a patient with oral submucous fibrosis and unknown diabetes mellitus. Peker et al. [6] and Kwak et al. [7] reported facial candidal abscess in uncontrolled diabetic patients. Of the 5 cases, 4 were associated with diabetes, and as in our case, these patients had high blood glucose levels. Diabetes mellitus is a metabolic disorder characterized by chronic hyperglycemia. Diabetic patients have an increased susceptibility to fungal infection including *Candida* species. Several alterations in host immune mechanisms have been described [11, 12]. The hyperglycemic condition may adversely affect the phagocytic activity of neutrophils, including migration impairment, phagocytosis, chemotaxis, and intracellular killing. In addition, compromised local circulation due to macro- and microvascular dysfunction may delay response to infection and impair wound healing.

Candidal infection of the skin and subcutaneous tissue may result from direct contact, inoculation injury (primary infection) or hematogenous spread (secondary infection) [13]. Several studies have demonstrated particular characteristics of *Candida* species that increase the incidence of candidiasis in diabetic patients [12]. *Candida* species exhibits higher hemolytic and esterase enzymatic activity in hyperglycemic condition, which may contribute to increased pathogenic capacity. High glucose levels are thought to provide a source of carbohydrate energy required for biofilm formation which provides protection against environmental challenges. Recently, Lin et al. [14] analyzed the incidence and risk factors of cellulitis following acupuncture treatment in Taiwan. The incidence of cellulitis was approximately 64 per 100,000 acupuncture treatments. Diabetes mellitus was significantly associated with an increased risk of cellulitis after acupuncture, and the risk was two times higher in diabetes patients than normal controls.

Acupuncture is a component of traditional Chinese medicine and has been widely used in East Asia to treat a variety of conditions including pain and swelling. Acupuncture is generally considered as a safe procedure with few complications when performed by qualified practitioners [15]. Despite guidelines for safe acupuncture practice [15], several case reports of adverse event following acupuncture have been reported. In this case, the patient punctured her eyelid using a non-disposable needle without an adequate skin disinfection and this may have caused an inoculation of pathogen. *C. albicans* on the skin may carry into the deep facial tissue through the needle or may invade through the skin defect and proliferate under conditions of impaired immune status due to uncontrolled diabetes. Strict sterile techniques such as a clean working environmental, clean hands, preparation of needling site, and sterile needle should be emphasized, and special attention is required when performing acupuncture in immunocompromised patients. The limitation of this case report is that details of acupoints, depth of insertion, and technique of acupuncture, which may be important information for the analysis of the cause of infection [16], are insufficient because the patient, who was not a health care professional, performed self-acupuncture at home.

The treatments for skin and subcutaneous fungal abscesses include surgical incision and drainage with a systemic antifungal agent. In our case, intravenous empirical antibiotics were started at first, however, the lesion progressed. Surgical incision and drainage was performed and *C. albicans* was isolated from the pus culture. Fluconazole is an azole antifungal agent widely used against *Candida* species. The patient finally recovered after several incision and drainages cycles, followed by systemic fluconazole treatment for 4 weeks, and her blood glucose level was well-controlled.

In summary, multiple facial *C. albicans* abscesses were eventually confirmed in our patient, thought to be caused by an acupuncture needle and undiagnosed diabetes mellitus. This case shows that unusual organisms and underlying immunocompromised conditions should be considered in cases with recurrent abscesses showing an inadequate response to initial antibiotic treatment. Culture and sensitivity tests are required to identify the specific organisms and ensure effective treatment. In addition, acupuncture should be performed with a sterile needle with an adequate skin disinfection by well-trained healthcare professionals.

## Data Availability

Not applicable.

## Abbreviations

*C. albicans*: *Candida albicans*
